# Supplementary material for: The aryl hydrocarbon receptor controls cyclin O to promote epithelial multiciliogenesis
Source: Nat Commun. 2016 Aug 24;7:12652. doi: 10.1038/ncomms12652 (PMC4999520; doi:10.1038/ncomms12652)
Supplement: Supplementary Information — Supplementary Figure 1-7 [file ncomms12652-s1.pdf]

Supplementary Figure 1

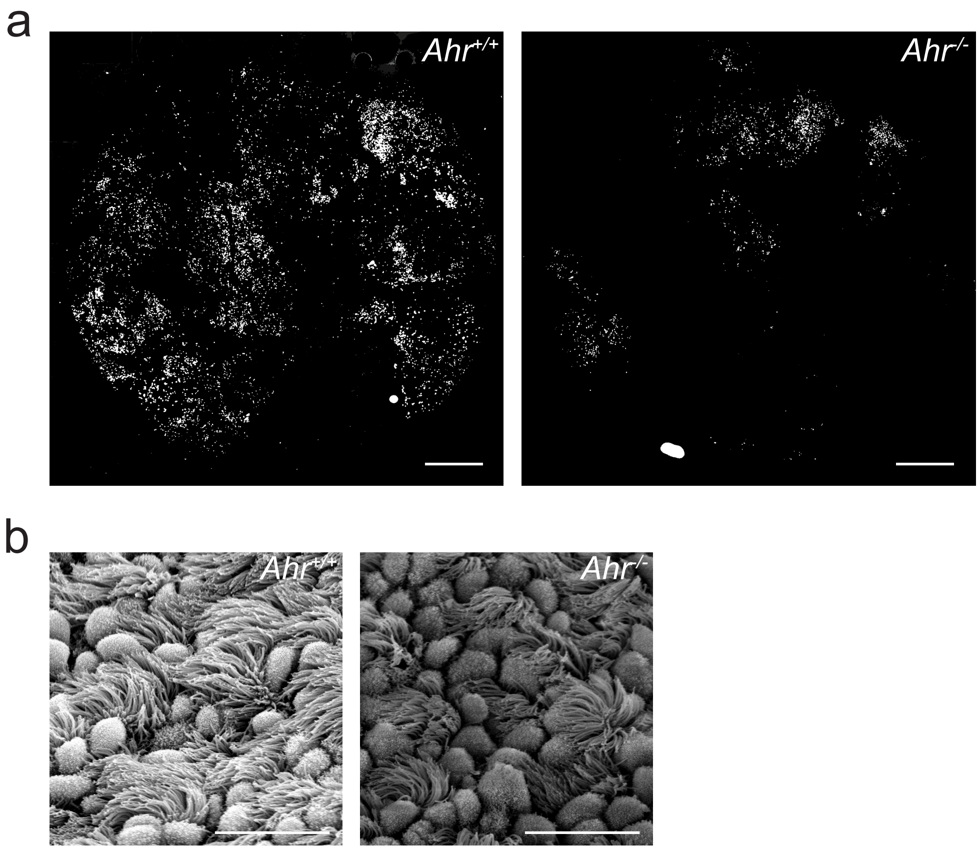

Supplementary figure 1 | The role of AhR in promoting ciliogenesis, not evident in adult mice, can be unveiled growing airway epithelial progenitors under ALI conditions. (**a**) Immunofluorescent staining of acetylated α-tubulin in AhR sufficient and AhR deficient mTEC cultures at 9 days of ALI. Representative images of the wells used for the quantification in figure 1c. Scale bars 1 mm. (**b**) Scanning electron microscopy (SEM) images of tracheae from 8 weeks-old AhR sufficient and AhR deficient mice. Scale bars 10 µm. Data representative n = 5 mice/group.

Supplementary Figure 2

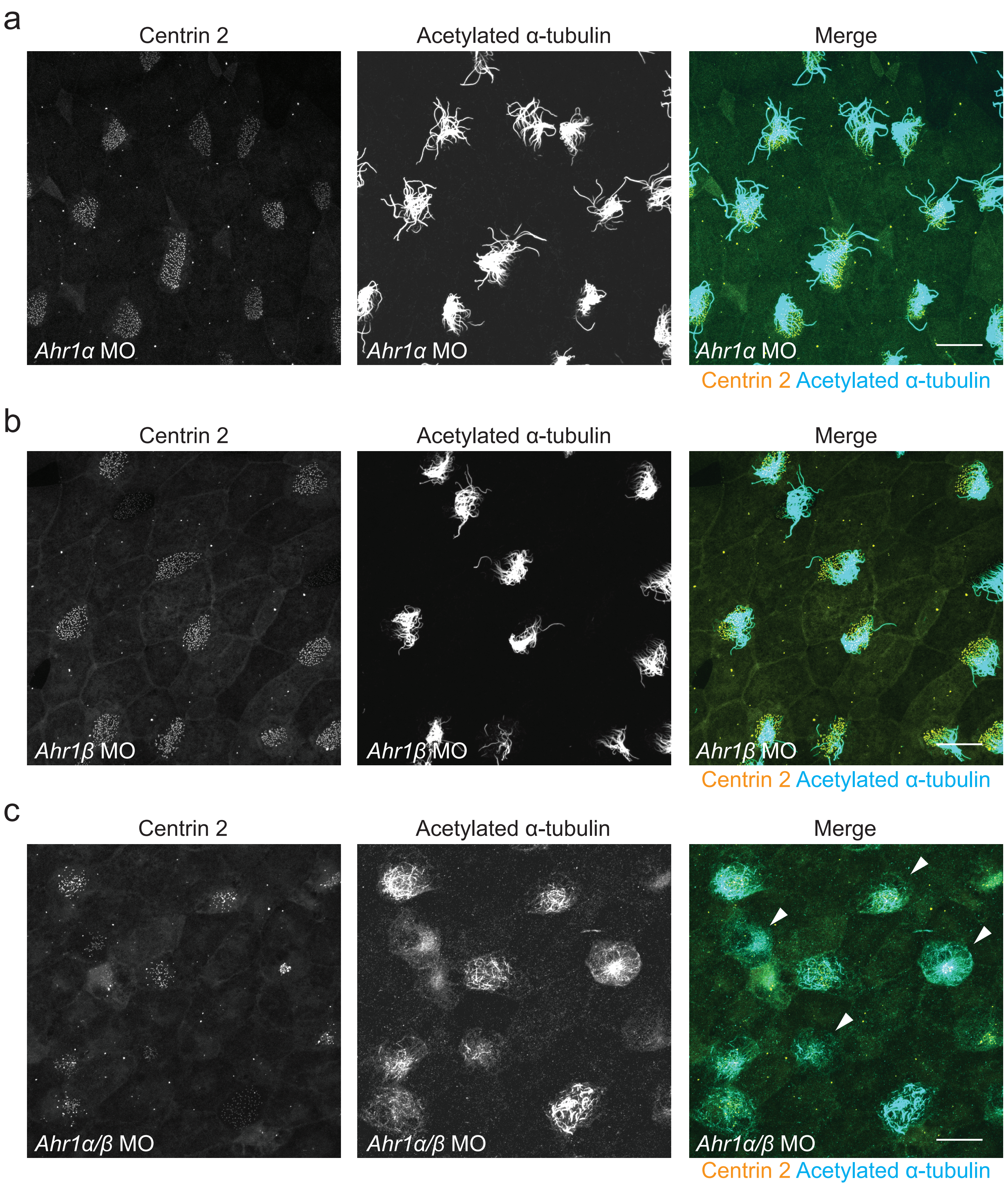

Supplementary figure 2 | Morpholinos targeting either *Ahr1α* or *Ahr1β* are not sufficient to impair ciliogenesis in *Xenopus laevis* embryos. (a-c) Immunofluorescent staining of acetylated α-tubulin (cilia, cyan) in the skin of embryonic *Xenopus laevis*, injected with either *Ahr1α* MO or *Ahr1β* MO or both. Injected cells are centrin 2^+ (centrin 2 localizes at the basal bodies, yellow). Arrowheads in (c) indicate examples of cells with disorganized ciliary pattern. Scale bars 20 µm.

Supplementary Figure 3

a

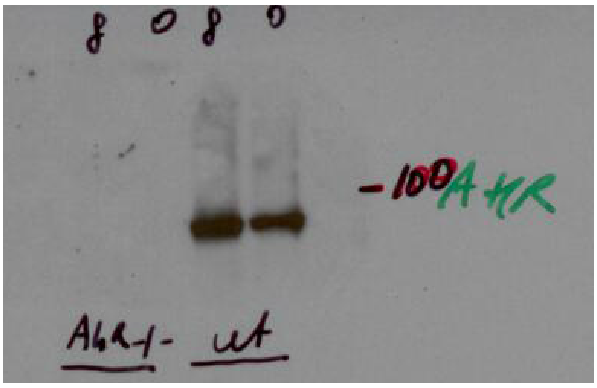

b

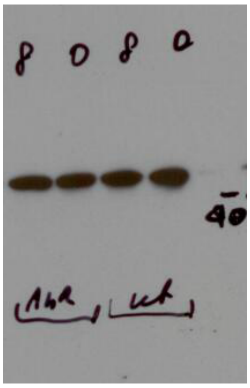

Supplementary figure 3 | Unmodified western blots shown in figure 4b. (a, b) Immunoblot analysis of AhR and β-actin protein expression, in AhR sufficient (wt) and AhR deficient mTEC cultures at days 0 and 8 of ALI. Data representative of three independent experiments.

Supplementary Figure 4

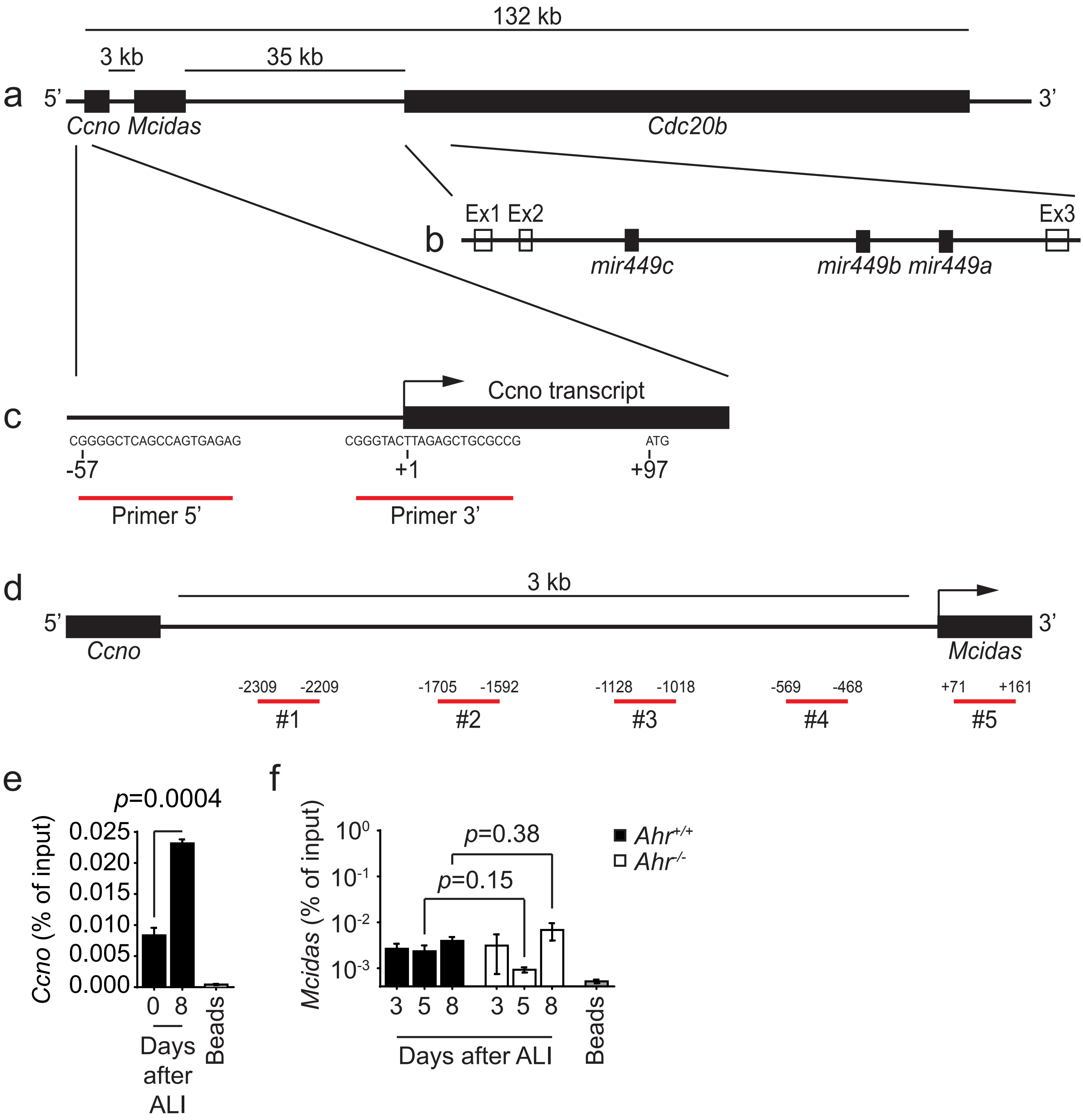

Supplementary figure 4 | AhR and ARNT collaborate in controlling *Ccno* expression. (**a**) Schematic overview of the portion of murine chromosome 13 spanning the genomic loci of *Ccno*, *Mcidas*, *Cdc20b* and *mir449*. (**b**) Diagram of the *Cdc20b* gene which contains *miR449a/c* in intron 2. (**c**) Schematic representation of the *Ccno* promoter region. Primers used for ChIP experiments are indicated. (**d**) Diagram of the 3kb intergenic region between the *Ccno* and the *Mcidas* genes. Amplicons analysed for ChIP experiments in (f) are indicated. (**e**) ChIP analysis of ARNT interaction with the *Ccno* promoter in AhR sufficient mTEC cultures at indicated days of ALI. Mean ± SEM; Student's t test (unpaired, two-tailed). Data representative of two independent experiments. (**f**) ChIP analysis (amplicon #4) of AhR interaction with the *Mcidas* promoter in AhR sufficient and AhR deficient mTEC cultures at indicated days of ALI. Similar results were obtained with the other amplicons depicted in (d). Mean ± SEM; Student's t test (unpaired, two-tailed). Data representative of three independent experiments.

Supplementary Figure 5

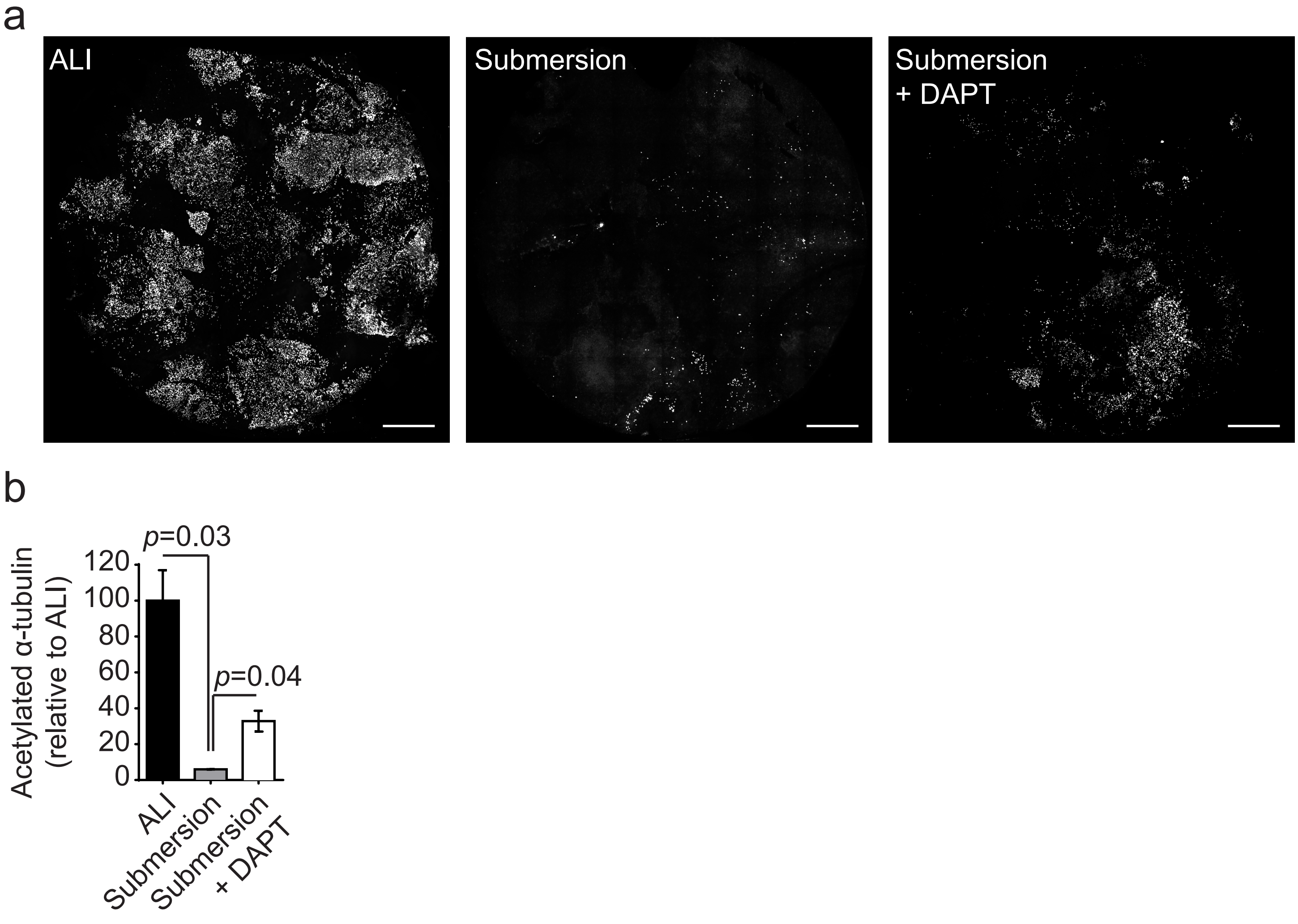

Supplementary figure 5 | Notch inhibition restores ciliated cell differentiation in submerged conditions. (**a**) Immunofluorescent staining of acetylated α-tubulin in AhR sufficient mTEC cultures grown in ALI, or submersion in presence or absence of DAPT. Representative images of the wells used for the quantification in (b). Scale bars 1 mm. (**b**) Quantification of acetylated α-tubulin fluorescence intensity from entire wells of AhR sufficient mTEC cultures grown in ALI, or submersion in presence or absence of DAPT. Fluorescence intensity of ALI condition was set as 100%. Mean ± SEM; Student's t test (unpaired, two-tailed). Data representative of two independent experiments.

Supplementary Figure 6

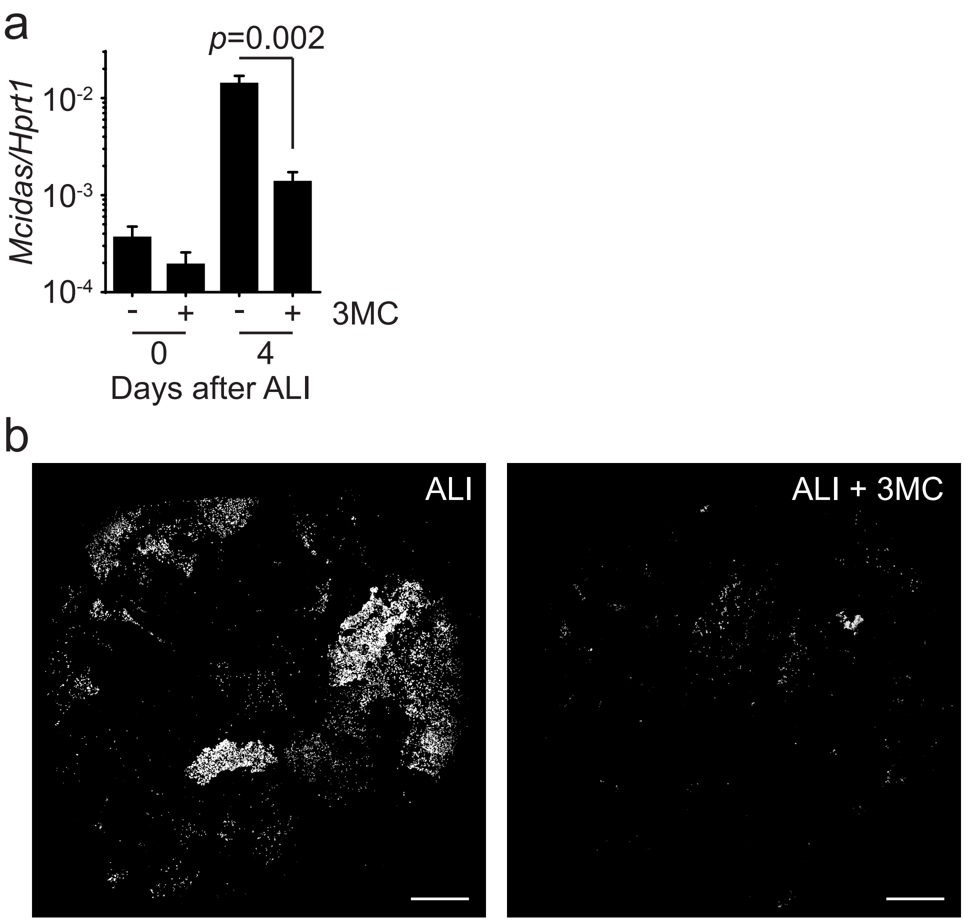

Supplementary figure 6 | AhR activation by environmental agents interferes with ciliogenesis. (**a**) mRNA expression levels of *Mcidas* in AhR sufficient mTEC cultures at indicated days of ALI and treated or not with 3 methylcholanthrene (3MC) starting 2 days before ALI onset. Values are normalized to *Hprt1*. Mean ± SEM; Student's t test (unpaired, two-tailed). Data representative of three independent experiments. (**b**) Immunofluorescent staining of acetylated α-tubulin in AhR sufficient mTEC cultures at 8 days of ALI in presence or absence of 3MC. Representative images of the wells used for the quantification in fig. 6d. Scale bars 1 mm.

Supplementary Figure 7

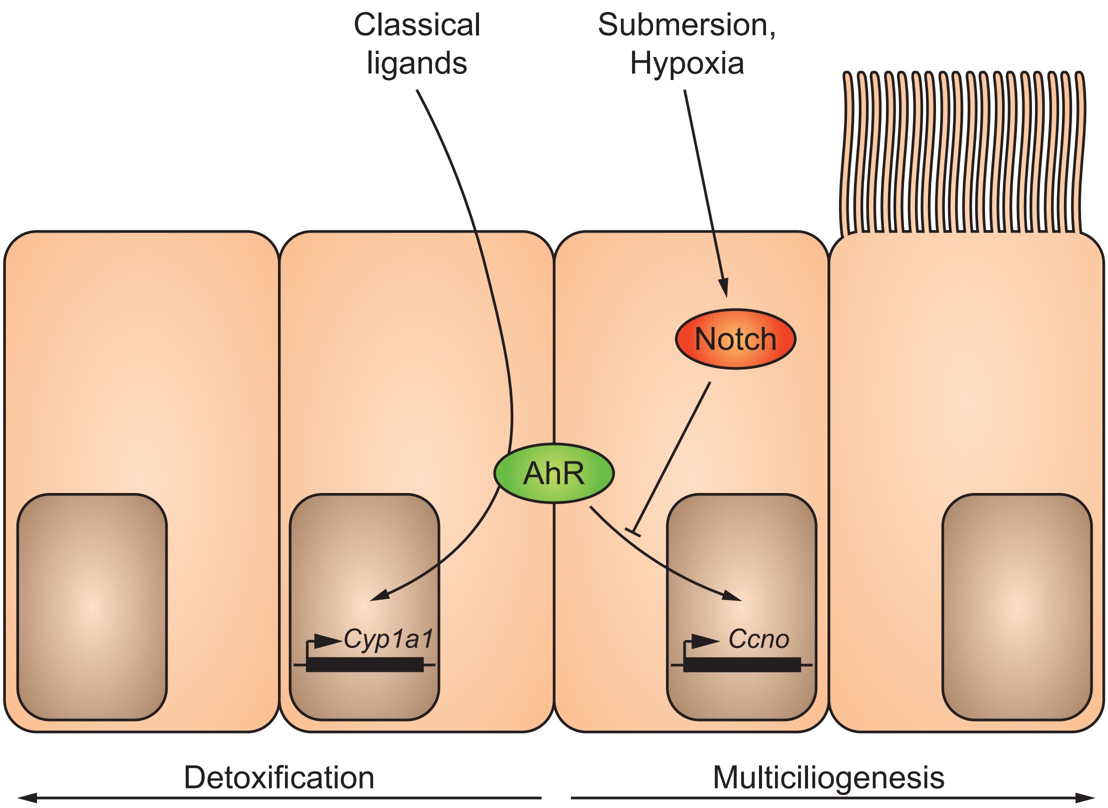

Supplementary figure 7 | Dual role of AhR in ligand-induced detoxification and in physiological ciliogenesis in multiciliated epithelial cells. AhR modulates both ciliogenesis and xenobiotic responses by driving differential gene transcription. Oxygen exposure counteracts ciliogenesis-inhibiting Notch signalling and licences AhR-dependent induction of *Ccno* and other targets involved in ciliogenesis. Classical AhR ligands can deviate AhR away from the physiological ciliogenesis pathway to induce transcription of genes involved in detoxification.
